# Contrast-enhanced ultrasound in critical care: a problem-solving approach

**DOI:** 10.3389/fmed.2026.1861070

**Published:** 2026-06-29

**Authors:** Lyu Yang, Wanhong Yin, Tongjuan Zou, Ran Zhou, Jun Li

**Affiliations:** 1 Visualized Diagnostics and Therapeutics & Artificial Intelligence Laboratory/Department of Critical Care Medicine, West China Hospital, Sichuan University, Chengdu, China; 2 Department of Critical Care Medicine, The First People's Hospital of Longquanyi District, Chengdu, China

**Keywords:** contrast-enhanced ultrasound, critical care, intervention, microbubbles, microcirculation, therapy

## Abstract

When the body enters a rapidly progressive and life-threatening pathological state, it is defined as critical illness. At this stage, respiratory and circulatory dysfunction are the most prevalent clinical manifestations. During disease progression and therapeutic interventions, persistent pathological insults, sustained microcirculatory hypoperfusion, and systemic inflammatory responses further contribute to complications such as coagulopathy, acute kidney injury, gastrointestinal dysfunction, and ICU-acquired weakness. Contrast-enhanced ultrasound (CEUS), as a non-invasive and bedside-applicable imaging modality, enables sensitive detection of tissue-level perfusion changes and provides crucial insights for the early identification of organ dysfunction and disease progression. Based on recent advances and our clinical experience, this article focuses on the evaluation and interventional strategies for perfusion-related pathophysiological processes in vulnerable organs within the ICU setting. In particular, we highlight the value of CEUS in assessing hemodynamics in the kidney, gastrointestinal tract, and skeletal muscle, emphasizing its role in microcirculatory monitoring, early disease recognition, CEUS-guided percutaneous interventions, and optimization of thrombus-related therapeutic decision-making. Collectively, these applications could support more precise interventions and have potential to improved patient outcomes.

## Introduction

In the clinical practice of critical care, ultrasound serves as the “eyes” of intensivists, providing comprehensive assessment of the structure, morphology, and hemodynamics of various organs and tissues. Due to its advantages, including no radiation, low cost, dynamic and repeatable imaging, and bedside applicability, ultrasound has been widely recognized and has become a routine tool in clinical practice. However, conventional ultrasound typically allows only static evaluation of large vascular circulation, which limits its ability to assess visceral organs and physiological or non-physiological cavities. CEUS utilizes microbubble contrast agents (UCAs), which, when exposed to a low-pressure environment in specific contrast modes, resonate and produce nonlinear signals, revealing true microvascular and macrovascular images. CEUS provides enhanced visualization of lesions (including viable and necrotic areas), helps define critical structures, plan procedural pathways, confirm the extent of cavities and their communication, determine procedural endpoints, and identify complications. By shortening the time required for final diagnosis, it reduces costs, risks, and the need for unnecessary follow-up computed tomography (CT) or magnetic resonance imaging (MRI) scans (1). This article is a narrative review focusing on the current and emerging applications of CEUS in critically ill patients. From a critical care perspective and informed by our clinical experience, we systematically summarize the available evidence and practical applications of CEUS in organ systems and pathophysiological processes commonly encountered in the intensive care unit (ICU). By integrating current literature with real-world clinical observations, this review aims to provide a practical framework for bedside implementation and to inform the design of future prospective studies in critical care CEUS. Relevant literature was identified through searches of PubMed, Embase, and Web of Science databases up to January 2026 using combinations of the following keywords: “contrast-enhanced ultrasound,” “CEUS,” “critical care,” “intensive care unit,” “microcirculation”, “acute kidney injury,” “intestinal ischemia,” “skeletal muscle perfusion,” “ultrasound-guided intervention,” and “sonothrombolysis.” Priority was given to clinical studies, translational research, consensus statements, and high-quality review articles relevant to bedside perfusion assessment and CEUS-guided interventions in critical illness. Additional references were identified through manual review of reference lists from relevant articles. In this review, we provide a concise overview of the application of CEUS in assessing organ perfusion in the kidney, gastrointestinal tract, and skeletal muscle. By characterizing tissue-level blood flow, CEUS could support the acquisition of early warning signals of disease progression, thereby may facilitate timely etiological interventions and has potential to improve the therapeutic responses, while also helping to avoid overtreatment. In addition, when combined with ultrasound, their unique acoustic properties offer novel insights into thrombus-targeted therapy. Although many of these applications remain investigational, they illustrate the expanding translational potential of CEUS in precision critical care.

### Principles, bedside applications, and advantages of CEUS in critical care

Critically ill patients frequently develop complex hemodynamic disturbances characterized by impaired tissue perfusion, microcirculatory dysfunction, and heterogeneous organ blood flow. Conventional imaging modalities and macrohemodynamic parameters often fail to adequately reflect tissue-level perfusion abnormalities. In this context, CEUS has emerged as a valuable bedside imaging technique capable of real-time assessment of organ microcirculation and tissue perfusion in critically ill patients. CEUS microbubble UCAs are stable during pulmonary circulation and, as blood pool agents, circulate to the capillary beds before being cleared via the pulmonary circulation, with the phospholipid shell metabolized in the liver. Consequently, there is no liver or kidney toxicity, and the incidence of fatal allergic reactions is as low as 0.001%, significantly lower than that of CT or MRI UCAs (2). Unlike conventional Doppler ultrasound, CEUS enables visualization of both macrovascular flow and microvascular perfusion in real time. Time–intensity curve (TIC) analysis further allows semi-quantitative assessment of tissue perfusion characteristics, including contrast arrival, peak enhancement, wash-in, and washout dynamics (3).

In critical care settings, CEUS can be performed repeatedly at the bedside without ionizing radiation or nephrotoxic contrast exposure, making it particularly suitable for hemodynamically unstable patients. CEUS has been increasingly applied to evaluate renal, intestinal, hepatic, and skeletal muscle perfusion, as well as to guide interventional procedures, detect active bleeding, assess thrombosis, and monitor treatment response. Furthermore, CEUS enables serial evaluation of perfusion changes over time, potentially supporting individualized hemodynamic optimization and early detection of evolving organ dysfunction. The following sections summarize current evidence and emerging applications of CEUS in the assessment of organ perfusion, invasive intervention, and thrombus-related therapy in critically ill patients. In this study, the ultrasound UCAs (UCAs) was administered using a bolus technique. The bolus technique produces a contrast enhanced image of the insonated region consisting of a wash-in phase, microvascular perfusion phase and venous wash-out phase. However, it should be noted that the bolus-transit technique likely to be confounded by cardiac output. In addition, the mixing of contrast with blood may not be uniform. Nevertheless, bolus contrast dominates the recent literature of CEUS. An alternative, albeit less well described but gradually gaining attention, technique utilizes an infusion of contrast and a destruction-replenishment model (4, 5).

### Contrast-enhanced ultrasound assessment of renal hemodynamics and morphology

Acute kidney injury (AKI) affects 30–60% of critically ill patients, leading to prolonged hospital stays, increased healthcare costs, and higher mortality rates (6, 7). In the ICU, the majority of AKI cases are due to the lower blood flow associated pattern, with the primary causes including sepsis, septic shock, decreased cardiac output, and fluid loss (8). In cases of cardiogenic or hypovolemic shock, where reduced cardiac output occurs, exogenous catecholamines used to treat shock may divert blood flow away from the mesenteric circulation, leading to more severe hypotension in visceral organs such as the kidneys (9, 10). Different vasopressors may exert different effects on renal blood flow. Mechanistic understanding of the effects of vasopressors on intra-renal perfusion remains incomplete, and the emerging imaging technique of CEUS may help further elucidate these mechanisms (11). CEUS is being used in clinical studies to evaluate vasopressors thought to have intrinsic renal haemodynamic effects (12). AKI, as a clinical syndrome, has a complex etiology, and the exact pathogenic mechanisms remain unclear. However, microcirculatory dysfunction is a key pathogenic mechanism associated with severe disease, making close monitoring of renal hemodynamics and timely optimization of blood flow essential for diagnosis and treatment. A clinical blind spot exists regarding whether optimizing the microcirculation can be achieved by restoring blood pressure and cardiac function to normal levels. Increasing evidence suggests that renal microcirculatory dysfunction may persist despite normalization of systemic hemodynamic parameters. Ultrasound allows for semi-quantitative grading of renal blood flow and provides an renal resistive index (RRI), which can serve as an initial screening tool for renal dysfunction (13). However, studies have found that the accuracy of these measurements is limited, and various influencing factors can lead to reduced sensitivity and specificity. CEUS, utilizing microbubble UCAs, visualizes microcirculation and quantifies blood flow by tracking the intensity of these microbubbles within the vasculature. This provides an accurate assessment of renal perfusion (14). A prospective longitudinal observational study found that CEUS assessment of renal cortical perfusion within 4 days of ICU admission in septic shock patients revealed that renal cortical hypoperfusion was not significantly related to the reduction of renal macro vessel blood flow or cardiac output, and it persisted as a characteristic feature in critically ill septic patients, and renal microcirculatory dysfunction lasted longer than that observed in large circulatory and other microcirculatory indicators (5). Using CEUS to visualize the organ microcirculation, regions of interest (ROIs) can be selected, and perfusion curves derived from intensity changes over time can be used to quantify renal microcirculatory blood flow changes. A meta-analysis showed that, before changes in urine output and serum creatinine occur in AKI patients, microcirculatory perfusion has already decreased, as reflected by prolonged perfusion time and decreased renal cortical ascending slope (AS) on CEUS (15). Thus, CEUS appears capable of detecting alterations in renal cortical perfusion before conventional markers such as serum creatinine or urine output become abnormal. CEUS can sensitively detect early changes in renal blood flow, even before clinical changes in creatinine and urine output are observed. In a lipopolysaccharide-induced septic shock pig model (16), it was found that despite normalization of mean arterial pressure, central venous pressure, and cardiac output, sublingual microcirculation measurements showed that fluid resuscitation caused microvascular occlusion, resulting in a decrease in functional capillary density. The same findings were observed in renal microcirculation, where CEUS demonstrated persistently reduced peak intensity, quantifying renal microcirculatory insufficiency. Using CEUS to monitor renal microcirculation may assist in identifying patients at risk of AKI and could provide additional information to support individualized hemodynamic management (17).

**Figure 1:**
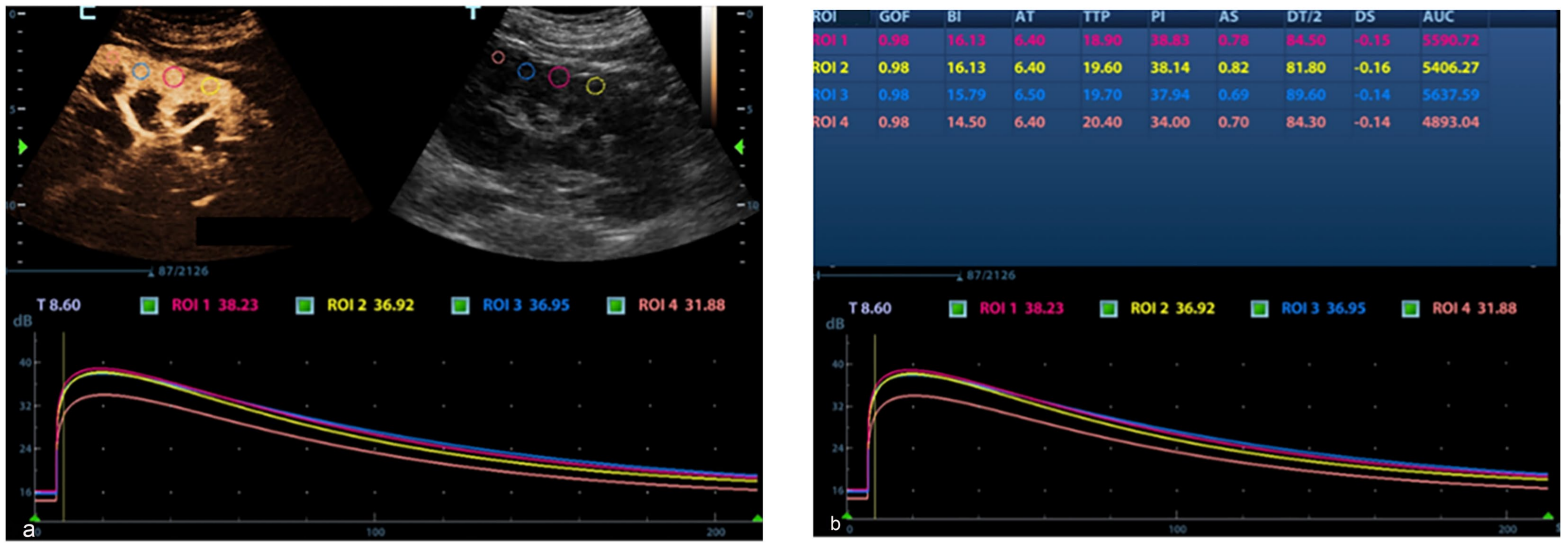
TIC of a normal renal contrast enhancement. **(a)** Cortical sampling site and corresponding TIC. The screen displays the contrast-enhanced image (C) alongside the conventional ultrasound image (T). **(b)** Plot of ROIs analyzed by dedicated software. The patient was a 46-year-old female diagnosed with pulmonary infection and respiratory failure, with normal serum creatinine and urine output. This is a normal renal CEUS image. These findings are consistent with preserved renal microcirculatory perfusion.

**Figure 2:**
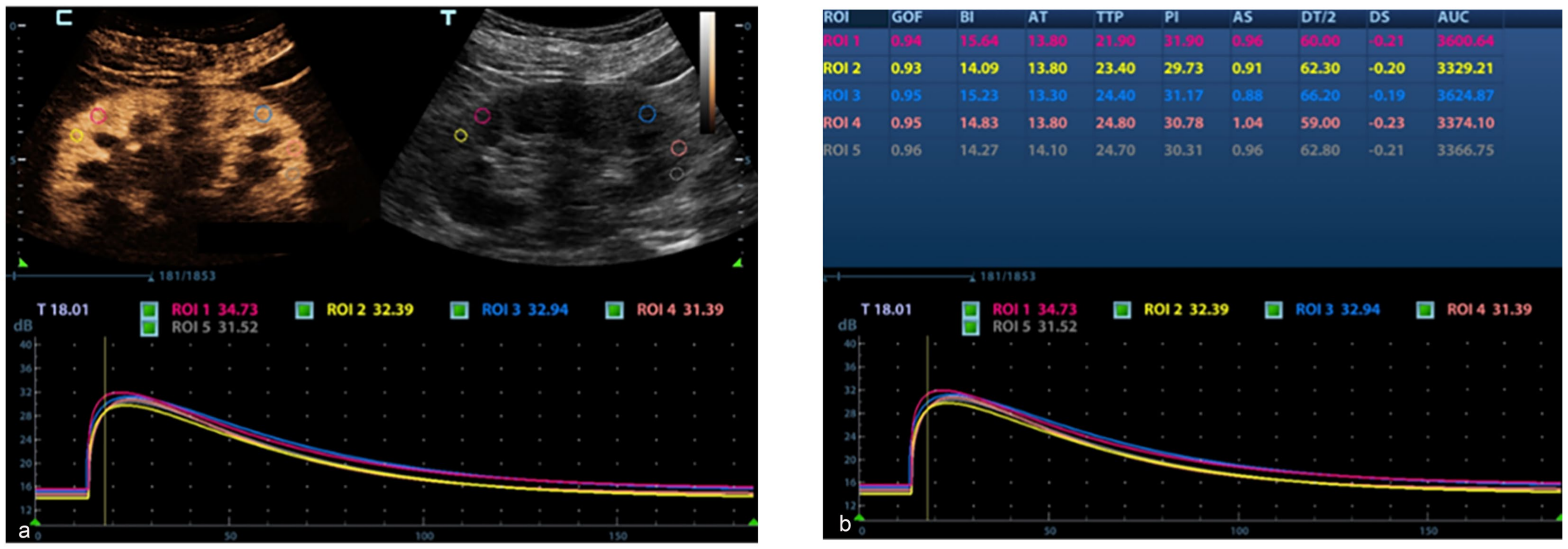
TIC of a forward flow insufficiency renal contrast enhancement. **(a)** Cortical sampling site and corresponding TIC. The screen displays the contrast-enhanced image (C) alongside the conventional ultrasound image (T). **(b)** Plot of ROIs analyzed by dedicated software, with fitted curves generated for each ROI (lower panel). The patient was a 64-year-old male diagnosed with intestinal obstruction and shock. CEUS parameters were characterized by prolonged AT and TTP,and a decreased AUC. This perfusion pattern may reflect delayed cortical microvascular inflow and reduced tissue perfusion, potentially suggesting hemodynamic hypoperfusion requiring early circulatory optimization.

**Figure 3:**
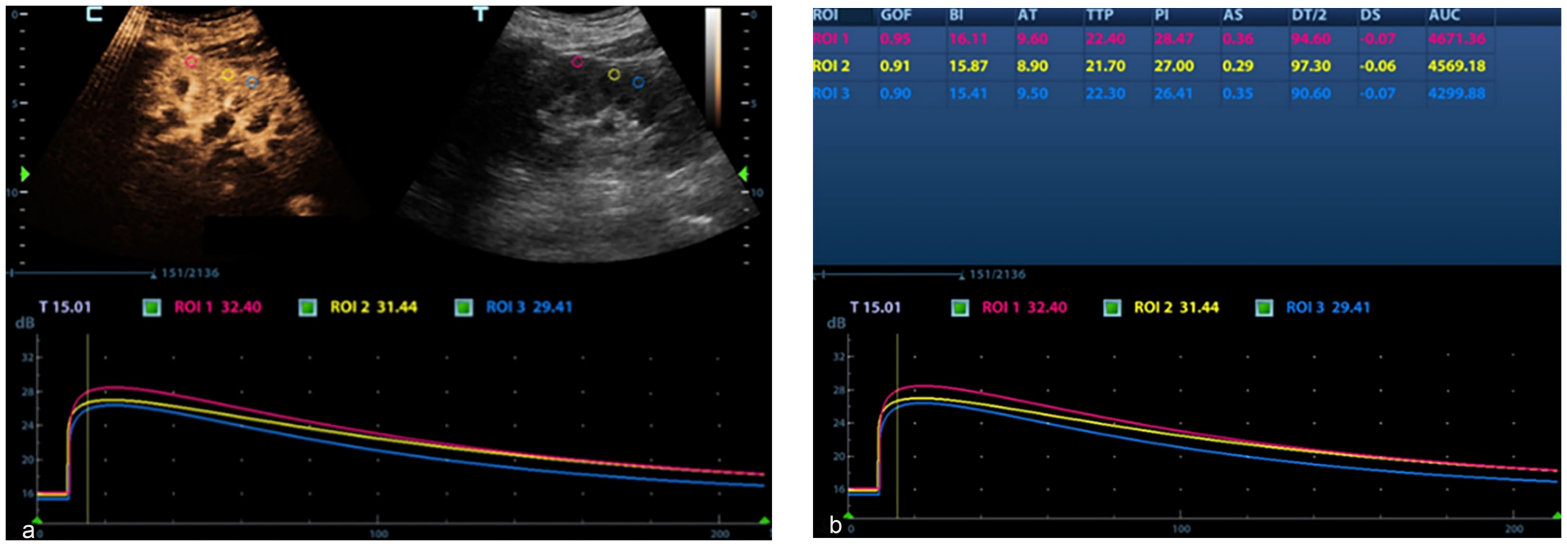
TIC of a reflux obstruction renal contrast enhancement. **(a)** Cortical sampling site and corresponding TIC. The screen displays the contrast-enhanced image (C) alongside the conventional ultrasound image (T). **(b)** Plot of ROIs analyzed by dedicated software. The patient was a 53-year-old male diagnosed with heart failure, pulmonary edema, and shock. CEUS parameters were characterized by markedly decreased AS and prolonged DT/2. These findings may indicate impaired venous outflow and prolonged microvascular washout, potentially associated with venous congestion–related renal perfusion abnormalities.

This is especially important in the context of the kidney, an encapsulated organ which may be adversely affected by increased venous tone. Accordingly, there is currently great interest in measuring venous flows both within the kidney and other organs as illustrated by the popularity of the Venous Excess Ultrasound Grading System (VExUS) in recent literature (3). Based on preliminary bedside CEUS observations, several representative renal perfusion patterns appeared to correspond to different hemodynamic states, including hypoperfusion-associated pattern, normal perfusion, and reflux obstruction patterns (Figures 1–3). These exploratory imaging patterns were descriptively characterized according to differences in TIC-derived parameters, such as prolonged AT/TTP with reduced enhancement in low-flow states and delayed washout in congestion-related states. Importantly, these observations should be interpreted as exploratory imaging phenotypes rather than a validated classification system, as they were derived from limited clinical cases without formal quantitative validation. The proposed patterns are not intended to represent universally applicable hemodynamic categories but rather imaging manifestations observed in selected clinical scenarios. Nevertheless, these preliminary findings suggest that CEUS may provide additional insights into renal microcirculatory alterations in critically ill patients and may serve as a basis for future prospective studies.

### Contrast-enhanced ultrasound assessment of intestinal hemodynamics and morphology

**Figure 4:**
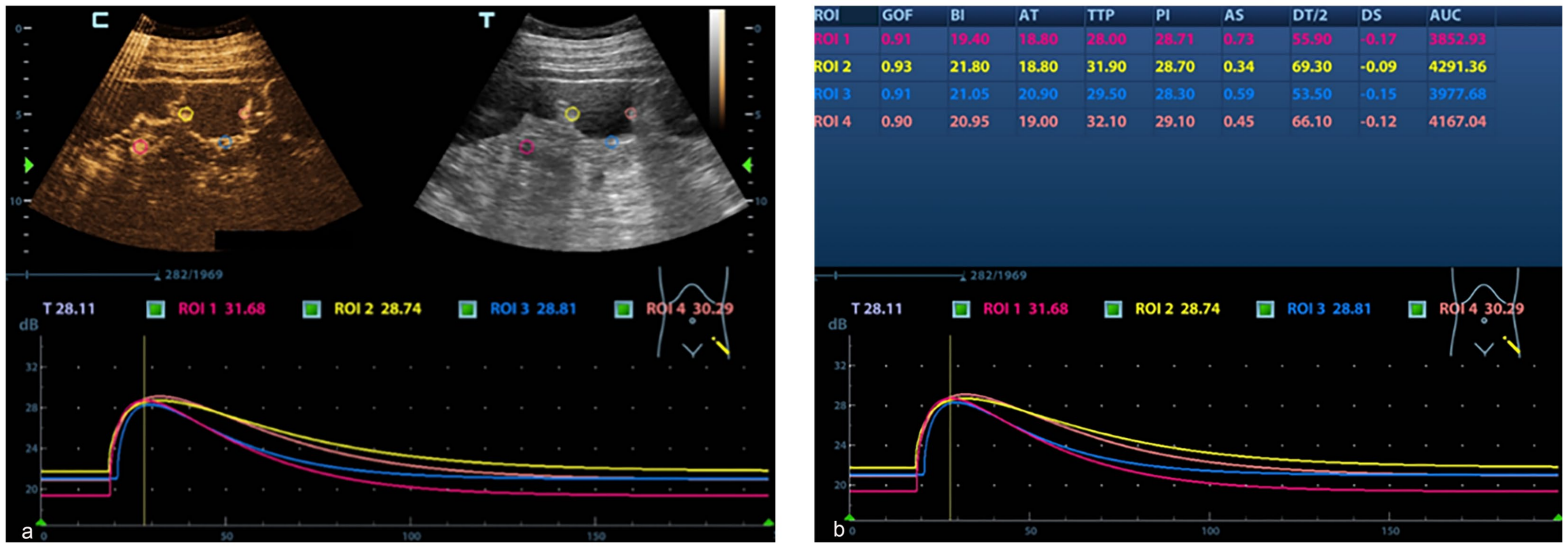
TIC of intestinal contrast enhancement with forward flow insufficiency type. **(a)** Bowel wall sampling site and corresponding TIC. The screen displays the contrast-enhanced image (C) alongside the conventional ultrasound image (T). **(b)** Plot of ROIs analyzed by dedicated software. The patient was a 40-year-old male diagnosed with traumatic intestinal rupture and mesenteric artery injury. CEUS parameters were characterized with a significant reduction in the AUC and prolonged TTP. This pattern may suggest impaired intestinal microcirculatory perfusion and delayed bowel wall enhancement, potentially indicating early ischemic injury.

**Figure 5:**
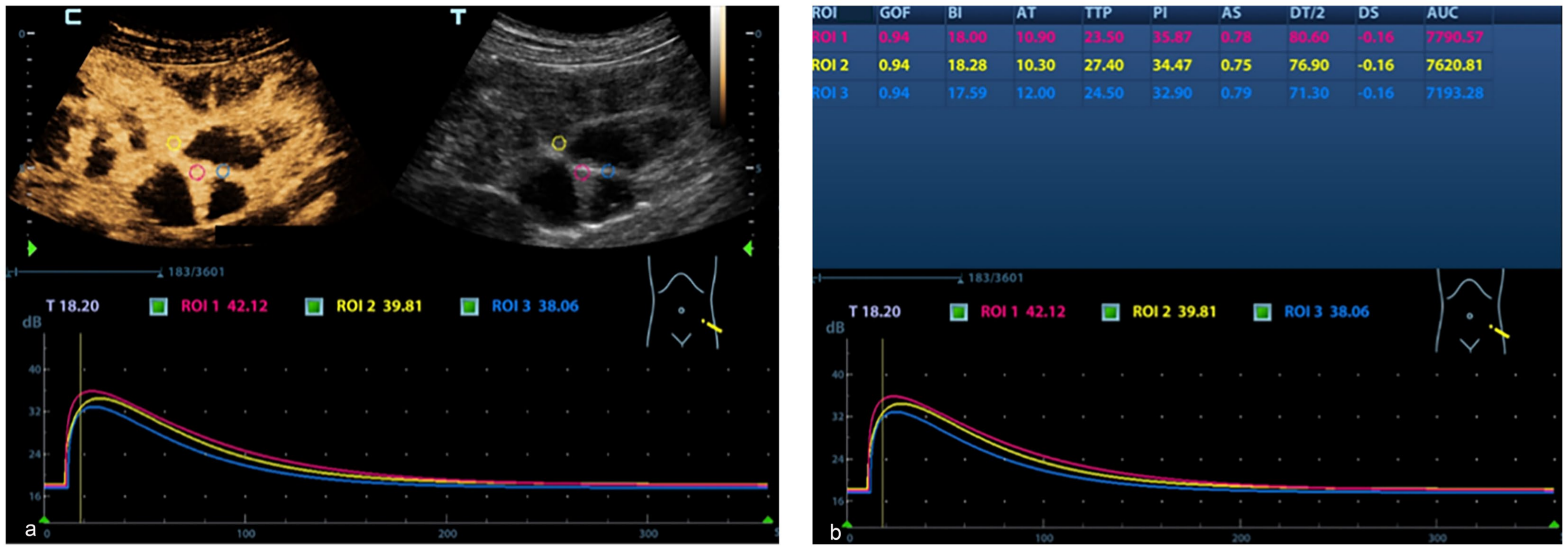
TIC of intestinal contrast enhancement with reflux obstruction type. **(a)** Bowel wall sampling site and corresponding TIC. The screen displays the contrast-enhanced image (C) alongside the conventional ultrasound image (T). **(b)** Plot of ROIs analyzed by dedicated software. The patient was a 30-year-old female with sepsis and volume overload. CEUS parameters were characterized by increased AUC and prolonged DT/2. These findings may reflect intestinal venous congestion and delayed contrast clearance, which may contribute to bowel wall edema and impaired perfusion.

Intestinal ischemia is a potentially life-threatening condition, and ischemia caused by shock, trauma, or sepsis can damage the gastrointestinal mucosa, representing one of the leading causes of death in patients with acute circulatory failure (18). Patients with impaired intestinal perfusion often have comorbidities and present with non-specific symptoms. Moreover, sedation and mechanical ventilation may mask many clinical features of intestinal injury, making timely diagnosis and treatment a major challenge. Untreated ischemia can progress to intestinal necrosis, with mortality rates reaching up to 50% (19). Surgical decision-making is time-sensitive and may be life-saving; however, inappropriate surgical intervention can lead to additional complications, particularly in hemodynamically unstable patients. Contrast-enhanced computed tomography (CECT) is the first-line imaging modality for suspected small bowel ischemia, but it has limited specificity. Although angiography is often considered the gold standard for definitive diagnosis, it is technically complex and not suitable for patients with only a suspicion of intestinal ischemia. In contrast, transabdominal ultrasound provides a non-invasive approach for gastrointestinal evaluation. Conventional Doppler ultrasound, however, may be inadequate for assessing microcirculatory perfusion in the intestine, as blood flow velocities and signal intensities often fall below the detection threshold of high-pass filters. CEUS, with high temporal resolution and real-time results, enables multi-planar imaging of localized regions of intestinal ischemia, making it a highly effective tool for early detection of small bowel ischemia. CEUS may offer high sensitivity in depicting ischemic changes, such as segmental bowel wall thickening with hypoechogenicity and loss of layered structure, which should always raise suspicion for ischemic disease (20). Furthermore, the enhanced spatial and temporal resolution of CEUS may allow detection of bleeding not visible on CT. For patients with suspected small intestinal obstruction based on CT findings, CEUS was performed. By sequentially displaying the microcirculation blood flow perfusion status of the intestinal wall, it was found that the sensitivity for detecting intestinal ischemia was 100%, the specificity was 85.7%, and the proportion of correct exclusion of intestinal wall ischemia was 91.7% (21). Currently, studies describing the role of CEUS in intestinal ischemia remain limited. We present two cases of patients with suspected intestinal ischemia and congestion, distinct CEUS perfusion exploratory imaging patterns may be observed under different hemodynamic conditions, including forward flow insufficiency (Figure 4) and reflux obstruction patterns (Figure 5). Early recognition of these perfusion abnormalities can guide interventions to maintain normal abdominal organ perfusion and reduce the risk of gut-derived infection. Future studies with larger cohorts of patients suspected of intestinal ischemia, combined with quantitative perfusion analysis, may help identify novel specific markers for non-invasive diagnosis and expand the clinical utility of CEUS in this context.

In addition to intestinal ischemia, active gastrointestinal (GI) bleeding is a common critical emergency. Although digestive endoscopy remains the first-line modality for identifying GI bleeding, the presence of large volumes of blood or stool can hinder adequate mucosal inspection. At high mechanical indices, CEUS microbubbles are disrupted by the ultrasound beam, whereas GI contents remain unaffected. By increasing the mechanical index, UCAs can be readily distinguished from GI contents, improving the sensitivity for detecting active rectal bleeding to 80% and for active diverticular bleeding to 50% (22). In patients with severe GI bleeding, jet-like or serpentine extravasation is often observed, whereas in less severe cases, extravasation tends to appear as round or oval foci. Moreover, CEUS allows continuous scanning of ROI, minimizing the risk of missing contrast agent extravasation due to delayed leakage from the vasculature. The ability of CEUS to detect active bleeding not only facilitates subsequent angiography and embolization procedures but can also be applied in postoperative patients with a high suspicion of intra-abdominal hemorrhage, thereby streamlining resuscitation and enabling early intervention (23, 24). For embolization of active bleeding, CEUS can be performed intra-procedurally via intra-arterial or catheter-directed contrast injection to confirm the endpoint of embolization.

### Contrast-enhanced ultrasound assessment of skeletal muscle hemodynamics and morphology

**Figure 6:**
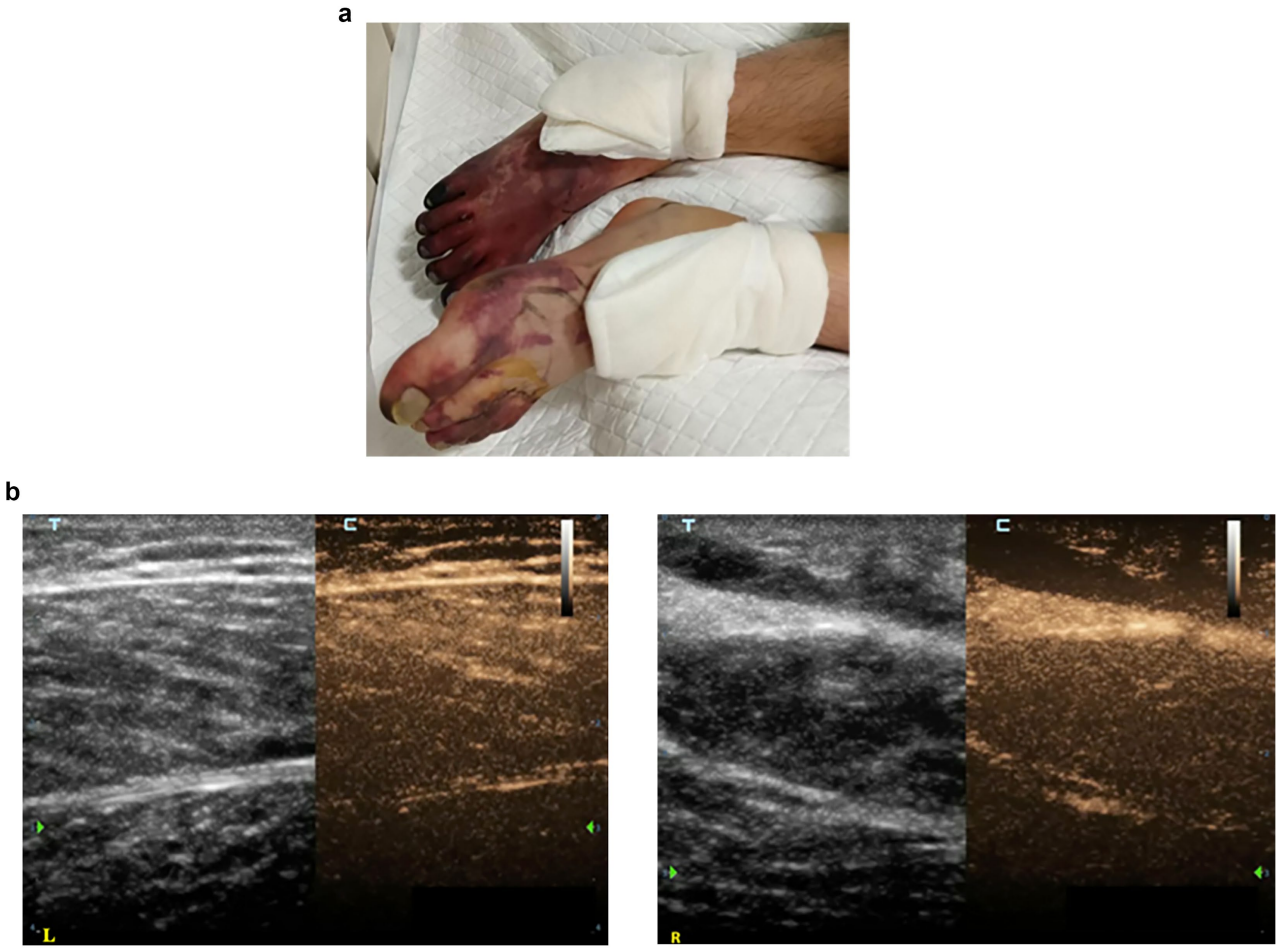
**(a)** An 18-year-old male presented with bilateral lower limb arterial thrombosis caused by prolonged kneeling, resulting in severe ischemic swelling, more pronounced on the right side. **(b)** The screen displays the contrast-enhanced image (C) alongside the conventional ultrasound image (T). L indicates the left side, and R indicates the right side. The two-dimensional ultrasound image shows disrupted and blurred muscle texture, with the right side (muscle swelling, loss of normal muscle texture, appearing hypoechoic) compared to the left side (clear muscle texture, natural and continuous orientation). CEUS reveals poor blood flow perfusion in the right gastrocnemius muscle. The reduced enhancement observed on CEUS may indicate severe skeletal muscle hypoperfusion and ongoing ischemic injury, potentially supporting early vascular or limb salvage intervention.

Similar to renal and intestinal perfusion, skeletal muscle microcirculation may also serve as a sensitive marker of systemic hemodynamic coherence in critically ill patients. Critically ill patients often experience prolonged hospital stays due to mechanical ventilation, sepsis, severe wounds, stroke, traumatic brain injury, and tracheostomy (25). Extended hospitalization predisposes these patients to secondary sarcopenia and ICU-acquired weakness (ICUAW). Reduced muscle blood flow, advanced age, underlying disease, decreased caloric intake, and elevated pro-inflammatory cytokines all contribute to the development of sarcopenia (26). From a pathophysiological perspective, microvascular injury and impaired nitric oxide production, leading to reduced muscle perfusion, are considered key mechanisms underlying muscle loss (27). Ultrasound provides a rapid and convenient method for assessing muscle mass, while CEUS enables real-time dynamic and quantitative analysis of skeletal muscle perfusion. By evaluating temporal changes in vascular bundle geometry and distribution on contrast-enhanced imaging, CEUS allows detailed characterization of blood flow and tissue perfusion in muscles and tendons. Weber et al. (28) quantified microvascular density using CEUS and compared perfusion parameters with histological vascularization from vastus lateralis muscle biopsies, demonstrating a strong correlation between the CEUS-derived parameter B and capillary fiber contacts, thereby supporting the utility of CEUS for quantitative assessment of skeletal muscle perfusion. Intravascular CEUS enables visualization and quantification of skeletal muscle microcirculation, allowing differentiation between muscle lesion and healthy controls, and can be used to monitor both quantitative and qualitative changes in the rectus femoris and quadriceps muscles in mechanically ventilated patients with suspected ICU-acquired weakness, with minimal adverse effects (29). Following contrast administration, areas with increased local perfusion or inflammatory exudation exhibit enhanced signal intensity, and CEUS can further depict augmented perfusion after transient arterial occlusion release (30, 31). Amarteifio et al. (32) further demonstrated that CEUS can assess skeletal muscle microvascular perfusion in patients with type 2 diabetes, with impairment correlating with disease duration. Hotfiel et al. (33). compared CEUS with conventional ultrasound and magnetic resonance imaging (MRI) in muscles with varying degrees of injury, showing that CEUS has superior sensitivity in the early detection of mild skeletal muscle lesions (including ultrastructural damage and muscle strain). CEUS allows clear visualization of skeletal muscle blood flow patterns and provides more accurate identification of intramuscular edema. By using gas-filled microbubble UCAs to visualize and quantify microcirculation within the capillary bed, CEUS can assess intramuscular perfusion in muscles such as the rectus femoris and vastus intermedius and help identify factors associated with reduced tissue perfusion, thereby having potential to facilitate early rehabilitation interventions (34). Mitchell et al. reported that CEUS using SonoVue, combined with intermittent high–mechanical index pulses to disrupt intravascular microbubbles, enables real-time measurement of microvascular blood volume and flow velocity in the quadriceps muscle (35). By improving limb perfusion, this approach may represent one potential mechanism through which could influence skeletal muscle health. CEUS has potential to demonstrate markedly reduced perfusion in the affected muscle, directly reflecting microcirculatory impairment (Figure 6). This finding underscores the added value of CEUS in detecting perfusion deficits beyond structural changes, thereby enabling more accurate assessment of skeletal muscle ischemia and supporting timely clinical decision-making.

Beyond structural assessment, CEUS-based evaluation of skeletal muscle perfusion may have important implications for critical care management. In critically ill patients, alterations in skeletal muscle microcirculation may serve as an early indicator of systemic perfusion abnormalities, vasopressor-related peripheral hypoperfusion, or the development of intensive care ICUAW. When interpreted in conjunction with vascular ultrasound and cardiopulmonary ultrasound findings, CEUS may provide additional clinical context for describing skeletal muscle perfusion characteristics. Such findings may offer insight into potential mechanisms of tissue hypoperfusion, including low cardiac output–related perfusion deficits, thrombus-associated impairment of arterial inflow, and venous congestion–related perfusion abnormalities. In selected clinical scenarios, CEUS findings may influence bedside decision-making by guiding fluid and vasoactive therapy, could support early identification of patients at risk for ischemic muscle injury or compartment syndrome, and prompting timely rehabilitation and nutritional interventions aimed at preserving muscle function.

### Contrast-enhanced ultrasound in cavitary structures and invasive procedures

CEUS has the potential to visualize both physiological and non-physiological cavities. Abscesses may occur in multiple locations, including the liver, spleen, kidneys, abdominal cavity, pleural space, mediastinum, bone, and soft tissues. Adequate drainage of abscesses is essential for source control, and timely intervention is required to effectively eliminate infection. The efficacy and safety of percutaneous abscess drainage have been widely recognized (36). Ultrasound-guided percutaneous drainage has become the preferred treatment modality due to the absence of radiation exposure and its high success rate (37). However, the management of fluid collections located in anatomically complex regions remains challenging. These collections are often deeply situated and obscured by overlying bowel or other organs, making it difficult to differentiate heterogeneous internal echoes of the lesion from adjacent intestinal structures. This limitation compromises ultrasound visualization and hinders the establishment of safe puncture pathways. Furthermore, complex fluid collections, such as those associated with pancreatitis, may appear nearly solid or mass-like, limiting their depiction on conventional two-dimensional ultrasound. In contrast, CEUS can demonstrate the absence of vascularity within abscess cavities and clearly delineate lesion boundaries as well as the surrounding enhancing parenchyma. This improves lesion characterization and helps minimize the risk of inadvertent injury to adjacent organs during percutaneous interventions. Although intracavitary administration of UCAs is currently considered an off-label application, several indications for intracavitary CEUS (IC-CEUS) have been reported in the latest EFSUMB guidelines on non-hepatic CEUS (38). CEUS provides additional benefits by delineating non-enhancing regions and adjacent critical vascular structures, distinguishing viable from non-viable tissues, and enabling retrograde contrast injection through indwelling catheters to confirm position and pathway (39–41). Taking pancreatitis as an example, CEUS can directly and clearly demonstrate the location and extent of peripancreatic abscesses, which appear as non-enhancing abscesses areas on ultrasound and correspond well with findings on contrast-enhanced CT, thereby improving the accuracy of lesion localization and characterization (42). Percutaneous drainage under imaging guidance can achieve effective fluid evacuation, which may contribute to improved source control (43, 44) and could support microbiological sampling to guide targeted therapy. Once access via a transmural or percutaneous route has been established, IC-CEUS can be performed using UCAs to assess the degree of communication between multiple cavities. This information is valuable for determining whether adjunctive interventions, such as thrombolysis or mechanical disruption, are required to achieve optimal drainage. Although tubes and drainage catheters can generally be identified on conventional B-mode imaging due to impedance differences, accurate localization along the entire catheter length or at the needle tip may be challenging. In contrast, IC-CEUS enables precise visualization of the needle or catheter position within the target area. Because the volume of intracavitary fluid is substantially smaller than the circulating blood volume, UCAs should be significantly diluted for intracavitary administration compared with intravascular use (45). *In vitro* studies have demonstrated that SonoVue remains stable in diluted form for up to 20–30 min (46). Furthermore, the use of high-energy ultrasound pulses to disrupt microbubbles allows for prolonged, dynamic, and repeatable intracavitary CEUS assessment. When contrast injection through the drainage catheter results in dispersion confined to the catheter tip, and forceful flushing leads only to localized accumulation of contrast agent, this suggests the presence of septations within the fluid collection. Such findings can guide the subsequent use of fibrinolytic therapy and assist in timely adjustment of catheter position to optimize drainage, as well as precise control of catheter withdrawal length (47, 48). By evaluating the distribution of intracavitary UCAs, IC-CEUS can assess communication between the abscess cavity and surrounding tissues or organs, thereby identifying complications such as fistula formation, bile leakage, or gallbladder perforation and may facilitate timely endoscopic or surgical intervention for definitive source control (39, 49). The clinical value of IC-CEUS in the management of abdominal fluid collections and abscesses exemplifies its role as a point-of-care ultrasound (POCUS) technique (39). In interventional imaging using UCAs, the contrast dose can be adjusted according to body mass index (BMI), lesion depth, and transducer frequency. During CEUS-guided interventions, multiple contrast injections may be required. An initial bolus injection is typically used to identify the target lesion and plan the procedure, while a second bolus (or, in some cases, continuous infusion) can be used to guide accurate placement of the puncture needle.

### Therapeutic applications of CEUS and microbubbles

Venous thromboembolism (VTE) in critically ill patients, including pulmonary embolism (PE) and deep vein thrombosis (DVT), is a major cause of unexpected in-hospital mortality (50). POCUS enables rapid and accurate assessment of various pathological conditions, including DVT, thereby facilitating timely bedside diagnosis and treatment (51, 52). Despite routine prophylactic anticoagulation with low-molecular-weight heparin (LMWH) or unfractionated heparin in critically ill patients, the incidence of VTE remains as high as 9.5% (53). This may be attributed to factors such as trauma or concomitant infection, which can interfere with the anticoagulant effects of heparin and lead to heparin resistance (54), as well as enhanced thrombin and fibrin generation, further increasing VTE risk (55). Thrombolytic therapy has notable limitations, including treatment failure and a risk of severe bleeding complications. Critically ill patients often receive multiple medications, making them more susceptible than non-ICU patients to subtherapeutic or excessive plasma drug concentrations (56). Regimens using recombinant tissue plasminogen activator (rt-PA) or tissue plasminogen activator are associated with a higher incidence of minor bleeding compared with urokinase, while high-dose thrombolytic protocols are frequently linked to severe hemorrhagic complications (57). Therefore, the development of safer and more effective anticoagulation and thrombolytic strategies remains a critical need. While CEUS has traditionally been used for vascular imaging, increasing attention has been directed toward the therapeutic potential of microbubbles. Under ultrasound stimulation, microbubbles exhibit unique acoustic and mechanical effects, including cavitation, shear stress amplification, and enhanced local drug delivery. These properties have expanded the role of CEUS from a purely diagnostic imaging modality to a potential theranostic platform integrating both imaging and targeted therapy. In this context, ultrasound–microbubble-assisted thrombolysis has emerged as a promising strategy for improving thrombus dissolution while potentially reducing systemic thrombolytic exposure and bleeding complications. Conventional ultrasound UCAs, limited by their micrometer-scale size, are confined to the intravascular space and have limited ability to detect extravascular lesions. In contrast, nanotechnology-based targeted UCAs offer advantages such as biocompatibility, controllable particle size, and stability in circulation. These agents can penetrate neovascular structures and, through surface-conjugated targeting ligands, have become a research focus for both diagnostic and therapeutic applications. Microbubbles loaded with thrombolytic agents can be driven into thrombi under low-frequency ultrasound, while high-frequency ultrasound induces cavitation within the thrombus. The combined effects of dual-frequency ultrasound enhance drug diffusion and cavitation, thereby improving thrombolytic efficacy. Previous studies have shown that microbubbles (MBs), when exposed to low-energy ultrasound, undergo resonance that amplifies shear and mechanical forces mediated by endothelial cells or erythrocytes in the microcirculation, increasing local tissue perfusion—a phenomenon known as “acoustic-assisted perfusion” (58, 59). Ultrasound-induced oscillation of MBs can increase endothelial nitric oxide synthase (eNOS) phosphorylation by up to sixfold, enhancing endogenous nitric oxide (NO) bioavailability via activation of the eNOS pathway and thereby improving blood perfusion (60, 61). Sawaguchi et al. (62) demonstrated in an *in vitro* clot lysis model that ultrasound combined with rt-PA increased thrombolytic efficiency by approximately 2.5-fold compared with rt-PA alone, while reducing the required rt-PA dose by 60%. The biological effects of ultrasound and microbubbles *in vivo* primarily occur at the endothelial surface or within deeper vascular wall structures, where their combined action disrupts thrombus architecture, facilitates deeper penetration of urokinase into the clot, accelerates fibrin degradation, enhances thrombolytic efficacy, and allows for reduced urokinase dosing with a lower risk of adverse events (63). In a validation study using a sheep model, dual-frequency ultrasound-assisted thrombolysis (DF-UAT) achieved iliac vein recanalization within 15 min (64). These findings support further investigation of DF-UAT as a potential adjunctive strategy for thrombus management. Furthermore, a multicenter randomized controlled trial evaluating ultrasound thrombolysis using low-intensity 2-MHz pulsed ultrasound during carotid endarterectomy demonstrated no increase in adverse events and a significant reduction in the composite endpoint of ischemic stroke, transient ischemic attack, and death within 30 days (65). These findings provide encouraging evidence regarding the feasibility and potential efficacy of ultrasound-assisted thrombolysis; however, further validation in larger clinical studies remains necessary. CEUS is evolving from a diagnostic imaging modality toward a theranostic platform. Looking forward, emerging applications such as drug-loaded microbubbles and sonoporation may further expand the therapeutic potential of CEUS in targeted thrombolysis and focal disease treatment. However, it should be emphasized that these approaches remain largely experimental and are currently supported primarily by preclinical and early translational studies. Further clinical validation is required before widespread implementation in routine critical care practice.

### Current limitations and challenges of CEUS in critical care

Although CEUS has gained increasing attention and demonstrated promising applications in critical care, several important limitations continue to restrict its widespread implementation and standardization in clinical practice. These limitations involve technical factors, operator dependency, quantitative variability, and the lack of large-scale clinical validation studies. Most therapeutic applications of CEUS and microbubbles remain at the preclinical or early translational stage and should not yet be considered established critical care interventions.

Despite these limitations, several strategies may improve the reliability and clinical applicability of CEUS in critical care. First, CEUS examinations should be performed and interpreted by appropriately trained operators with expertise in both critical care ultrasonography and contrast-enhanced imaging. In current clinical practice and research settings, image acquisition and quantitative analysis are typically conducted according to standardized protocols, incorporating internal quality control procedures, external quality assurance programs, and systematic review of image quality. Importantly, CEUS findings are rarely interpreted in isolation. Rather, they are integrated with conventional ultrasound, Doppler parameters, laboratory results, hemodynamic data, and, when available, complementary imaging modalities such as CT and MRI. Such multimodal correlation has become an established approach for improving diagnostic confidence and reducing interpretation bias. Acoustic window limitations remain a recognized challenge for all ultrasound-based techniques. However, CEUS may partially overcome some of the constraints encountered with conventional B-mode and Doppler imaging. The strong nonlinear signal generated by intravascular microbubbles improves lesion conspicuity and enhances visualization of tissue perfusion, even in situations where grayscale imaging is suboptimal. Consequently, CEUS often provides diagnostically useful information in patients with complex anatomy, postoperative changes, or suboptimal baseline sonographic images, although image quality may still be affected by severe obesity, extensive bowel gas, or deep-seated lesions. Another important limitation is the lack of universally accepted perfusion thresholds for defining organ hypoperfusion, congestion, or other perfusion phenotypes in critically ill patients. At present, most CEUS-derived quantitative parameters are interpreted within the clinical context rather than against validated cutoff values. Future progress will depend on prospective studies with standardized acquisition and analysis protocols. Large multicenter investigations are particularly needed to establish reproducible reference ranges, validate clinically relevant perfusion patterns, and determine outcome-associated thresholds. Several collaborative research initiatives are currently underway to collect multicenter CEUS datasets, which may provide a stronger evidence base for future perfusion classification systems and facilitate broader clinical implementation (Table 1).

**Table 1:** Main component parameters of TIC.

Parameter	Meaning	Physiological interpretation
AT	Arrival time	Blood flow velocity
TTP	Time to peak	microvascular filling speed
PI	Peak intensity	Blood volume
RT	Rise time	wash-in efficiency
AUC	area under the curve	Total perfusion
AS	Ascending slope	Perfusion speed
DT/2	Descending time/2	venous drainage efficiency

## Conclusion

CEUS is increasingly recognized as a valuable bedside imaging modality in critical care medicine, with growing applications in the assessment of organ perfusion, microcirculatory abnormalities, invasive interventions, and thrombus-related disorders. In critically ill patients, CEUS enables real-time visualization of both macrovascular and microvascular perfusion without ionizing radiation or nephrotoxic contrast exposure, making it particularly suitable for dynamic bedside monitoring in hemodynamically unstable patients. Current evidence suggests that CEUS may provide additional information beyond conventional hemodynamic assessment by facilitating evaluation of renal, intestinal, and skeletal muscle perfusion, detection of active bleeding, characterization of cavitary lesions, and guidance of interventional procedures. Furthermore, emerging applications involving ultrasound-responsive microbubbles and targeted thrombolytic strategies highlight the evolving translational potential of CEUS-based technologies.

However, several important limitations remain. With further technical standardization and clinical research, the integration of CEUS with advanced perfusion analysis, and Doppler-based hemodynamic assessment, may has potential to facilitate earlier recognition of tissue hypoperfusion, individualized bedside management, and more precise critical care interventions.
